# Construction of a plant-transformation-competent BIBAC library and genome sequence analysis of polyploid Upland cotton (*Gossypium hirsutum* L.)

**DOI:** 10.1186/1471-2164-14-208

**Published:** 2013-03-28

**Authors:** Mi-Kyung Lee, Yang Zhang, Meiping Zhang, Mark Goebel, Hee Jin Kim, Barbara A Triplett, David M Stelly, Hong-Bin Zhang

**Affiliations:** 1 Department of Soil and Crop Sciences, 2474 TAMU, Texas A&M University, College Station, TX, 77843-2474, USA; 2 College of Life Science, Jilin Agricultural University, Changchun, Jilin, China; 3 USDA-ARS, Southern Regional Research Center, Cotton Fiber Bioscience, 1100 Robert E. Lee Blvd, New Orleans, LA, 70124, USA

**Keywords:** BIBAC library, *Gossypium hirsutum*, *Gossypium raimondii*, BIBAC end sequence (BES), Genome evolution, SSR, Polyploidization and evolution

## Abstract

**Background:**

Cotton, one of the world’s leading crops, is important to the world’s textile and energy industries, and is a model species for studies of plant polyploidization, cellulose biosynthesis and cell wall biogenesis. Here, we report the construction of a plant-transformation-competent binary bacterial artificial chromosome (BIBAC) library and comparative genome sequence analysis of polyploid Upland cotton (*Gossypium hirsutum* L.) with one of its diploid putative progenitor species, *G. raimondii* Ulbr.

**Results:**

We constructed the cotton BIBAC library in a vector competent for high-molecular-weight DNA transformation in different plant species through either *Agrobacterium* or particle bombardment. The library contains 76,800 clones with an average insert size of 135 kb, providing an approximate 99% probability of obtaining at least one positive clone from the library using a single-copy probe. The quality and utility of the library were verified by identifying BIBACs containing genes important for fiber development, fiber cellulose biosynthesis, seed fatty acid metabolism, cotton-nematode interaction, and bacterial blight resistance. In order to gain an insight into the Upland cotton genome and its relationship with *G. raimondii*, we sequenced nearly 10,000 BIBAC ends (BESs) randomly selected from the library, generating approximately one BES for every 250 kb along the Upland cotton genome. The retroelement *Gypsy/DIRS1* family predominates in the Upland cotton genome, accounting for over 77% of all transposable elements. From the BESs, we identified 1,269 simple sequence repeats (SSRs), of which 1,006 were new, thus providing additional markers for cotton genome research. Surprisingly, comparative sequence analysis showed that Upland cotton is much more diverged from *G. raimondii* at the genomic sequence level than expected. There seems to be no significant difference between the relationships of the Upland cotton D- and A-subgenomes with the *G. raimondii* genome, even though *G. raimondii* contains a D genome (D_5_).

**Conclusions:**

The library represents the first BIBAC library in cotton and related species, thus providing tools useful for integrative physical mapping, large-scale genome sequencing and large-scale functional analysis of the Upland cotton genome. Comparative sequence analysis provides insights into the Upland cotton genome, and a possible mechanism underlying the divergence and evolution of polyploid Upland cotton from its diploid putative progenitor species, *G. raimondii*.

## Background

Cottons (*Gossypium* L. species) are the leading fiber and an important oilseed crop in the world. Cotton fibers sustain the world’s textile industry and are an alternative of the synthetic fibers whose production annually consumes billions of barrels of fossil oil worldwide. Cottonseeds are traditionally used to produce food oil and currently have been used as the feedstock of biodiesel production. Furthermore, cottons are also a model system for studies of plant polyploidization, cellulose biosynthesis and cell wall biogenesis. The cultivated cottons, Upland cotton (*G. hirsutum* L.) and Sea Island cotton (*G. barbadanse* L.), are allotetraploids containing two homoeologous genomes defined A- and D-subgenomes. It was estimated that they originated from their diploid progenitor species about 1–2 million years ago [1-3]. Therefore, the cotton polyploid-diploid complex has been widely used as a model system for study of plant polyploidization and its impacts on speciation, biology and evolution [4,5]. Cotton fibers, usually 20–40 mm long and 15 μm thick, are derived from individual epidermal cells of developing seeds and more than 90% of their content is cellulose. Cellulose is a major component of plant cell walls and constitutes the largest portion of plant biomass, with an estimated annual world production of billions of metric tons. Therefore, cotton research is of significance not only for the world’s textile and energy industries, but also for understanding the mechanism underlying cellulose biosynthesis and cell wall biogenesis [6-9] that are applicable to the entire plant kingdom.

Cottons have been subjected to extensive research in modern genomics and genetics [10]. A number of molecular genetic maps and several thousands of DNA markers have been developed, hundreds of genes and QTLs (quantitative trait loci) of agronomic importance have been mapped, and a large collection of expressed sequence tags (ESTs) have been generated from a variety of tissues collected at different growth and development stages [10]. Bacterial artificial chromosome (BAC) libraries have been demonstrated to be crucial for different aspects of advanced genomics and genetics research, and have been constructed for some genotypes of Upland cotton [11-14] and Sea Island cotton [15]. A draft physical map [16] and draft genome sequences (http://www.ncbi.nlm.nih.gov/sra/SRA024364?report = full) have been generated recently for *G. raimondii*. *Gossypium raimondii* contains a D genome (D_5_) and was proposed to be the closest diploid progenitor of the D-subgenome of allotetraploid cottons, including Upland cotton [4]. Nevertheless, no large-insert plant-transformation-competent binary BAC (BIBAC) library has been reported yet for any of the cotton species. BIBAC libraries have all of the functionality of BAC libraries, but also can be used as a vehicle of large DNA fragments containing a number of genes and QTLs for direct transformation in plants *via* either *Agrobacterium*[17-21] or biolistic bombardment [22] because they have their own selection markers for plant transformation. The direct transformation competence of BIBACs streamlines their uses for gene and QTL cloning [23], large-scale genome functional analysis [19,22,24] and molecular breeding. For instance, using BIBACs spanning the interval of a QTL (e.g., 5 – 10 cM), the DNA fragment containing the QTL could be identified by BIBAC transformation, without the need of high-resolution mapping used in the traditional but laborious process of map-based cloning. Using BIBACs with an average insert size of about 150 kb as a vehicle, the Upland cotton genome could be directly transformed into another genome by approximately 16,000 transformations, which is within the realm of current transformation technologies [17,19,22]. These utilities of BIBACs will dramatically facilitate many aspects of current genomics and genetics research such as gene and QTL cloning, targeted marker development and molecular breeding. Moreover, transformation of BIBACs with an insert size of about 150 kb potentially containing 5–30 genes allows transferring in a single event a cluster of genes that are likely involved in a particular biological process, thus improving the efficiency of molecular breeding [22,24]. Finally and importantly, transformation of large-insert BIBACs is particularly desirable for engineering genes from a species to an unrelated species, such as from cotton to *Arabidopsis,* because the native regulatory sequences and neighboring genes of the donor species are important to the transgene expression in the recipient species [22,24]. Therefore, a BIBAC library constructed from a cultivated cotton is of significance for cotton gene and QTL cloning [23], genome functional analysis and molecular breeding [22,24].

The genome origin and polyploidization of polyploid cottons, including two cultivated cottons (*G. hirsutum* and *G. barbadense*) and three wild species (*G. tomentosum*, *G. mustelinum* and *G. darwinii*), have been studied extensively [1-5]. A few extant diploid species containing an A or a D genome, including *G. raimondii*, have been proposed to be the most closest reluted of the polyploid cotton A- and D-subgenomes [4]. Nevertheless, debate widely exists about the genome origin and evolution of the polyploid cotton A- and D-subgenomes because such knowledge is of significance for enhanced cotton genetic improvement. The recent release of the *G. raimondii* draft genome sequence (http://www.ncbi.nlm.nih.gov/sra/SRA024364?report = full) provides useful tools for advanced studies of the origin and evolution of the polyploid cotton A- and D-subgenomes.

In this study, we constructed a large-insert BIBAC library from Upland cotton (*G. hirsutum*) cv. “Texas Marker-1” (TM-1) having a genome size of 2,425 Mb/1C [25], sequenced the ends of a large number of the BIBACs and preliminarily compared the genome of Upland cotton with the D genome of *G. raimondii*. Upland cotton represents approximately 95% of cotton growing in the world, and the genotype TM-1 has been widely used in cotton genomics and genetics research [14]. Therefore, the BIBAC library constructed from the genotype provides tools and resources for many aspects of cotton genomics, genetics and molecular biology research. From the BIBAC library, we isolated the BIBAC clones containing a number of genes of agronomic importance, such as those involved in fiber development (*MYBB*, *MYBT2* and *RDL1*), fiber cellulose biosynthesis (*CelA1*, *CelA3*, *CelA6*, *GhCesA2*, *GhIRX3*, *GhCesA3,* and an unnamed *Ces*), seed fatty acid metabolism (*FADO6*) and host-nematode interaction (*MIC3* and *MIC1-15*). Moreover, we also isolated the BIBAC clones potentially containing the genes conferring resistance to cotton bacterial blight pathogen, *Xanthomonas campestris* pv. *malvacearum*. Finally, we sequenced approximately 10,000 BIBAC ends (BESs) randomly selected from the library, with a sequence sample of 400 bp in an average of every 250 kb along the Upland cotton genome, to provide some insights into the Upland cotton genome and its relationships with *G. raimondii*. Using the BESs, we estimated the composition of the Upland cotton cv. TM-1 genome, identified over 1,269 SSRs, of which 1,006 were new, and compared the genome sequence of Upland cotton with that of *G. raimondii*. These results significantly promote genomics and genetics research of Upland cotton, including integrative physical and genetic mapping, genome sequencing and functional analysis of the Upland cotton genome, and provide deeper insights into the mechanisms underlying the genome origin, variation and evolution of Upland cotton.

## Results

### Construction of the BIBAC library

We successfully constructed a high-quality BIBAC library for Upland cotton using the TM-1 megabase-sized nuclear DNA partially digested with *Bam*H I (Invitrogen, Carlsbad, CA) in pCLD04541 [26,27], a widely-used, *Agrobacterium*-mediated plant-transformation-competent BIBAC vector [22,28-35]. Cottons are extremely abundant in polyphenolics [36,37] that may interact with DNA, thus interfering with DNA digestion and cloning [36,38,39]. Therefore, we prepared the DNA plugs from the cotyledons collected from the seedlings growing in a controllable growth chamber and treated in dark for 48 h (see the Methods). These measures minimized the contents of metabolites in the cells, especially polyphenolics and polysaccharides that may significantly influence the cloning of large DNA fragments. Furthermore, we washed the nuclei two more times than the original procedure for megabase-sized DNA preparation [37,39,40]. This measure further minimized the chloroplasts, mitochondria, polyphenolics and polysaccharides contained in the cytoplasm. Therefore, the resulting agarose plugs of the cotton nuclear DNA were transparent and pale in color, which is generally considered to be well suited for large-insert DNA library construction [38]. The third measure that we took to enhance the quality of the BIBAC library was the preparation of the DNA plugs at a proper concentration of DNA per plug. This is because the concentration of DNA in the plugs would significantly affect the methods and results of size selection on agarose gels, thus the insert sizes of the BIBAC clones resulted from the DNA [38]. Therefore, the use of the tissues containing reduced amount of polyphenolics and polysaccharides plus additional washes of nuclei before embedding into LMP agarose plugs is an effective and economical method to enhance the quality of megabase-sized DNA and ultimately the quality of the resultant BIBAC library. Preparation of the source DNA with an appropriate concentration in the agarose plugs further improved the average insert size of the resultant BIBAC library.

### Characterization of the BIBAC library

**Figure 1:**
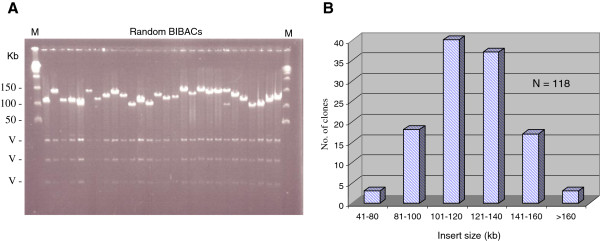
**Analysis of BIBACs randomly selected from the Upland cotton cv. TM-1 BIBAC library.** The BIBACs were digested with *Not* I and fractionated on a pulsed-field gel (**A**) and plotted according to their insert sizes (**B**). M, lambda ladder PFG marker; V, vector fragment; N, number of random clones analyzed for insert size estimation of the library.

The clones of the Upland cotton cv. TM-1 BIBAC library were individually arrayed into 200 384-well microtiter plates, containing a total of 76,800 clones. To determine the insert sizes of the clones and the portion of insert-empty clones in the library, we selected a random sample of 122 clones from the library, isolated DNA from the clones, digested with *Not* I (Invitrogen) and analyzed on pulsed-field gels (Figure 1A). Four of the 122 clones (3.3%) had no insert and the remaining 118 (96.7%) all had inserts. The distribution of the clones according to their insert sizes is shown in Figure 1B. The clones had insert sizes ranging from 60 to 250 kb, with an average insert size of 135 kb. Of the clones, nearly 80% had insert sizes larger than 100 kb. Furthermore, we estimated the percentage of the clones in the library derived from chloroplast DNA. A total of 691 positive clones were identified, accounting for approximately 0.9% of the library clones. Therefore, when the insert-empty clones and the chloroplast DNA-derived clones were excluded, approximately 95.8% (73,575) of the library clones contained inserts derived from cotton nuclear DNA. Since Upland cotton cv. TM-1 was estimated to have a genome size of 2,425 Mb/1C [25], the BIBAC library had a 4.1× coverage of the TM-1 haploid genome, with a 98.4% probability of obtaining at least one positive clone from the library using a single-copy probe [41,42].

### Identification of BIBACs containing genes important in cotton

To further characterize the library and test its utility for cotton genome research, we screened the library with 13 genes of agronomic importance (Additional file 1: Table S1), and four RGAs (resistance gene analogues) closely linked to the cotton bacterial blight resistance (Table 1). The library screening with the 13 genes resulted in a total of 60 positive clones, with each gene probe having 2–8 positive clones and an average number of 4.6 positive clones (Table 2), suggesting that the BIBAC library is suitable for map-based cloning and characterization of genes and QTLs important in cotton. The library screening with the four RGAs yielded 1, 72 and 104 positive clones for 1C08, 2D14 and 2D03/2B19, respectively (Table 1). Since the genome coverage of the library was 4.1 ×, the sequences of 2D14 and 2D03/2B19 are possibly dispersed in as many as 17 and 25 loci, respectively, in the Upland cotton genome.

**Table 1:** **Positive clones of the Upland cotton TM-1 BIBAC library identified by library screening with RGAs closely linked with the resistance to bacterial blight pathogen, *Xanthomonas campestris *pv. *malvacearum***[43,44]

**2D03 (AY600401) and 2B19 (AY600394) (104 positive clones):**					
B004B14	B011P19	B013N15	B017B02	B017H07	B019I04
B025M21	B026F24	B030I22	B041O11	B046C16	B046H18
B048P23	B050K21	B051D16	B051D23	B052F10	B055I19
B059G06	B059N17	B060P23	B061B24	B062I17	B063I15
B063L01	B065M06	B068H24	B069J11	B071B09	B071D22
B072P14	B075A15	B076D08	B080N11	B085C16	B085D09
B085G19	B087G17	B089E06	B091L11	B092C13	B092P12
B093J03	B097P19	B104A01	B105F15	B105G23	B108L23
B108P19	B110J23	B111C17	B111K03	B112G15	B112K13
B117K22	B118M16	B119B09	B120H01	B121B01	B125O02
B133A14	B134N15	B135C10	B136C02	B136O19	B138A12
B138A18	B138D03	B139E13	B140B05	B140J23	B145L08
B147F15	B148P17	B151K12	B155E18	B160G13	B161M18
B162E05	B162H06	B163B09	B165D12	B169M17	B170D18
B171A18	B175N17	B177F17	B177M15	B178C14	B179B04
B179D06	B179J12	B179N18	B184A18	B186F07	B188D16
B188E15	B188G13	B188I15	B189B12	B189J03	B192O18
B197K02	B199N18				
**2D14 (AY600383) (72 positive clones):**					
B006G23	B007N18	B008N22	B017B02	B017K11	B022M07
B037I22	B015L11	B016M13	B013N15	B014N21	B015O20
B011P19	B025B03	B032B23	B028D04	B030D15	B026F24
B031I22	B042J09	B047M01	B045O16	B048P23	B050F06
B049K06	B050K21	B052L03	B055P20	B071D19	B069E09
B069F11	B069K14	B070M07	B066O02	B068P02	B085C16
B085G19	B057I10	B061L07	B059M03	B061O06	B061O22
B075A15	B080E20	B077L10	B075N13	B073N21	B096D03
B093J03	B100J14	B115E13	B117K23	B120M11	B115P05
B133J15	B107D12	B111K03	B142J06	B148M10	B166A18
B165D12	B163H11	B161M18	B184A18	B181A23	B183D05
B179O02	B154N23	B171A18	B175N14	B176O01	B198J14
**1C08 (AY600376) (1 positive clone):**					
B125E12					

**Table 2:** The positive BIBAC clones of the genes identified by screening the Upland cotton cv. TM-1 BIBAC library using overgo probes designed from the gene sequences

**Gene**	**Positive clones**	**No. of clones**
*CelA1*	B130C07, B016N19	2
*CelA3*	B024E21, B072G17, B086A07, B083I01, B073K01, B073L02, B096F08	7
CelA6	B098I23, B116B12, B017I05, B035E07, B115K16, B005M05, B086H21	7
*MIC3*	B099A19, B024H22, B014D05, B027E13, B026F22, B092E14	6
*MIC1-15*	B099A19, B024H22, B014D05, B027E13, B026F22, B092E14	6
*RDL1*	B175F03, B050B20, B187C03, B186M07, B016L07, B016L07, B166C05, B161G09	8
*FADO6*	B138P05, B080A19	2
*MYBB*	B174C01, B192C23	2
*MYBT2*	B026F22, B007F03	2
*GhCe1A2*	B046G15,B048N17, B085P21, B162F12	4
*GhIRX3*	B070C20, B173F02	2
*GhCelA3*	B009G10, B065P05, B075L23, B097O20, B108L10, B146H15, B170C06, B178M15	8
Unnamed *Ces*	B008D22, B145L18, B164E04, B165A23	4

### BES analysis

A total of 10,752 BIBAC ends randomly selected from the Upland cotton BIBAC library were sequenced. After base calling and trimming the *E. coli* and vector sequences, 9,711 (90.3% successful rate) Q20 BESs with a minimum length of 50 bp were generated (dbGSS JY253441-JY262664). The total length of BESs was 3,842,009 bp, with each BES having a sequence length ranging from 50 to 842 bp, with an average length of 395 bp. Of these 9,711 BESs, 4,441 BIBAC clones (84.3%) were successfully sequenced at both ends, generating mate-paired reads. Sequence analysis of the BESs indicated that the Upland cotton cv. TM-1 genome, as many other dicotyledonous plant genomes, is A/T-rich with 64.50% A/T and 35.50% G/C (Table 3). From the 9,711 BESs, we identified a total of 2,912 or 3,022 exons, depending on the computer programs used (Table 3), and a total of 374,019 base pairs of repeated sequences. Of the transposable elements identified, the retroelement *Gypsy/DIRS1* family alone accounted for 77.86% (4.22/5.42) (Table 4).

**Table 3:** Summary of the Upland cotton BIBAC end sequences (BESs) generated and their exon and SSR contents

Total BESs sequenced	10,752
Q20 (50–842 bp)	9,711
Paired-ends	4,441
GC level	35.50%
Total length	3,842,009 bp
Average read of BESs	395.63 bp
Number of exons:	
GenScan	3,022
GeneMark	2,912
Microsatellite (di, tri, tetra, penta and hexa):	
Four or more repeat units	1,269
Five or more repeat units	313
Six or more repeat units	103

**Table 4:** Characteristics of repeat elements contained in the Upland cotton BIBAC end sequences (BESs)

**Elements**	**Number of elements**	**Length occupied (bp)**	**Percentage of the BES (%)**
Retroelements	776	208,081	5.42
LINEs:	21	4,469	0.12
*L1/CIN4*	21	4,469	0.12
LTR elements:	755	203,612	5.39
*Ty1/Copia*	166	40,878	1.06
*Gypsy/DIRS1*	578	162,030	4.22
DNA transposons	5	302	0.01
MuDR-IS905	3	152	0.00
Total interspersed repeats	208,383	5.42	
Small RNA	131	36,531	0.95
Simple repeats	201	10,777	0.28
Low complexity	2,289	118,328	3.08

### Identification of microsatellites from the BESs

**Figure 2:**
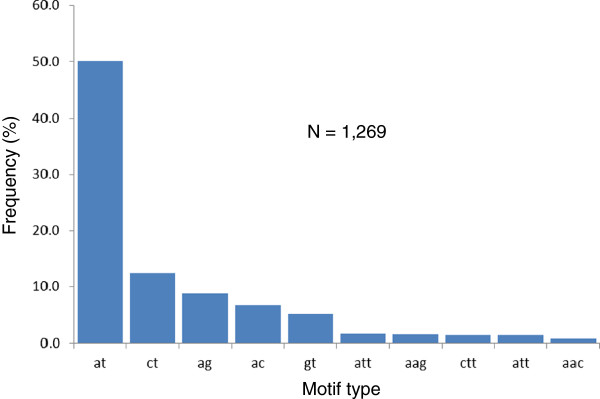
**Frequency of the ten most abundant SSR motifs in the Upland cotton genome.** The SSR motifs are di-, tri-, tetra-, penta- and hexa-mers, with four or more repeats.

To identify new SSR markers from the BESs, we analyzed the BESs for SSRs and compared the SSRs identified in this study with the existing cotton SSR database (http://www.cottonmarker.org). We identified a total of 1,269, 313 and 103 SSRs having motifs di-, tri-, tetra-, penta- and hexa-mers under the settings of ≥ 4, ≥ 5 and ≥ 6 repeat units, respectively (Table 3). Analysis of the 1,269 SSRs showed that dimer SSRs absolutely predominate in the Upland cotton genome (Figure 2). Among the dimer SSR motifs, (AT)_n_ (where *n* ≥ 4) was the most abundant in the genome, accounting for nearly 50% of all SSRs in the genome, and (CT)_n_, (AG)_n_, (AC)_n_ and (GT)_n_ were after (AT)_n_ in descending order, with each accounting for from 12.5% down to 5.5%. The other types of SSR motifs together only accounted for approximately 15% of the SSRs in the genome. To determine how many of them were new, we downloaded the cotton SSR database that contains 17,343 SSRs from the Cotton Marker Database (http://www.cottonmarker.org). The 1,269 SSRs identified in this study were then searched against the cotton SSR database. As a result, the locus sequences of 263 of the 1,269 SSRs matched the cotton SSR database; therefore, 1,006 or 79.28% of the 1,269 SSRs were novel.

### Comparative sequence analysis of the polyploid Upland cotton genome with the diploid *G. raimondii* genome

The origin of the D-subgenome of tetraploid cottons, including Upland cotton and Sea Island cotton, remains obscure, even though *G. raimondii* was previously proposed to be the closest diploid progenitor [4,45]. Because the whole genome sequence of *G. raimondii* has recently become available to the public, we compared the Upland cotton genome with the *G. raimondii* genome using the 9,711 BESs generated in this study as inquiries (Table 5). Surprisingly, only 57 of the 9,711 (0.59%) Upland cotton BESs were aligned to the *raimondii* genome using the criteria of a continuous minimal match of 100 bp and a sequence identity of 100%. This number corresponded to only 0.41% of the total sequence length of the BESs attempted in the alignment. When the sequence identity was reduced to 90% or lower while the criterion of continuous minimal match of 100 bp was maintained, the percentage of the Upland cotton BESs aligned to the *raimondii* genome reached and stabilized at approximately 70%, corresponding to approximately 51% of the total inquired Upland cotton BES sequence length.

**Table 5:** **Comparative sequence analysis of Upland cotton BESs with the D genome sequence of *G. raimondii *(DOE Joint Genome Institute, Cotton D V1.0,**http:// http://www.phytozome.net/cotton.php **)**

**Inquired upland cotton BESs**	**Criteria of the analysis**	**Sequence similarity**					
	Continuous MinMatch (bp)	100	100	100	100	100	100
All AD-subgenome BESs	Sequence identity (%)	100	95	90	80	70	60
(No. of BESs = 9,711; total sequence length = 3,842,009 bp)	No. of BESs aligned	57	4,587	6,588	7,118	7,257	7,277
	% of BESs aligned	0.59	47.24	67.84	73.30	74.73	74.94
	Total sequence length of BESs aligned to the *raimondii* sequence (bp)	15,539	1,367,385	1,960,413	2,048,311	1,996,820	1,975,511
	% of sequence length of BESs aligned to the *raimondii* sequence	0.41	35.59	51.10	53.31	51.95	51.42
	Continuous MinMatch (bp)	100	100	100	100	100	100
A-subgenome-specific BESs	Sequence identity (%)	100	95	90	80	70	60
(No. of BESs = 418; total sequence length = 169,533 bp)	No. of BESs aligned	7	205	294	310	317	317
	% of BESs aligned	1.67	49.04	70.33	74.16	75.84	75.84
	Continuous MinMatch (bp)	100	100	100	100	100	100
D-subgenome-specific BESs	Sequence identity (%)	100	95	90	80	70	60
(No. of BESs = 184; total sequence length = 71,410 bp)	No. of BESs aligned	1	88	128	136	139	139
	% of BESs aligned	0.54	47.83	69.57	73.91	75.54	75.54

Moreover, we extracted the Upland cotton A- and D-subgenome specific BESs from the 9,711 BESs using the A- or D-subgenome-derived BIBACs identified by Zhang et al. [46]. We were able to identify a total of 418 BESs derived from the A-subgenome BIBACs and 184 BESs derived from the D-subgenome BIBACs of Upland cotton (Table 5). When the A- and D-subgenome specific BESs were separately aligned to the *G. raimondii* genome sequence using the same criterion as above, the percentages of aligned BESs were not different from those obtained using the 9,711 BESs as inquiries. This result suggested that the genomic regions of Upland cotton from which the A- and D-subgenome specific BIBACs were derived have similar sequence identities to the other genomic regions of the A- and D-subgenomes with the *G. raimondii* genome. Since *G. raimondii* only has a D genome (D_5_), it was expected that the percentage of the D-subgenome specific BESs aligned to the *G. rainmodii* genome would be much higher than that of the A-subgenome specific BESs. Surprisingly, no significant difference was obtained between the percentages of the A- and D-subgenome specific BESs aligned to the *G. raimondii* genome.

## Discussion

We have developed a large-insert BIBAC library for Upland cotton in a BIBAC vector (pCLD04541) that is competent for direct plant transformation *via* both *Agrobacterium*[29,30] and biolistic bombardment [22]. The library contains 76,800 clones, with an average insert size of 135 kb. Therefore, the Upland cotton BIBAC library has a reasonably large average insert size. The 76,800 clones of the BIBAC library, when the insert-empty clones and the clones derived from chloroplast DNA are excluded, provide a 4.1× coverage of the Upland cotton haploid genome, with an approximate 99% probability of obtaining at least one positive clone from the library using a single-copy sequence probe. The results from the library screening with 13 gene-specific overgo probes designed from the unique sequences of genes, in which we obtained 2–8 positive clones for every probe, support the estimated genome coverage of the library, even though some of the probes might hybridize to two or more copies of the sequences because Upland cotton is an allotetraploid containing two homoeologous genomes, A- and D-subgenomes. Importantly, differing from the existing cotton BAC libraries [11-15], this BIBAC library is the first one competent for direct plant transformation *via* both *Agrobacterium*[29,30] and biolistic bombardment [22]. The plant transformability of the BIBAC library streamlines the map-based cloning of cotton genes and QTLs (Introduction) and large-scale functional analysis of the cotton genome through high-throughput BIBAC transformation [22,24]. Therefore, the BIBAC library reported here will promote many aspects of cotton genome research, including map-based gene and QTL cloning, genome physical mapping, genome sequencing and functional analysis of the Upland cotton genome.

We have used the BIBAC library to isolate BIBACs containing or closely linked to the genes controlling fiber development, fiber cellulose biosynthesis, seed fatty acid metabolism, cotton-nematode interaction, and cotton bacterial blight resistance. This experiment has not only further verified the quality and demonstrated the utility of the BIBAC library for cotton genomics research, but also provided the tools essential for characterization of the genes at the genomic level and promoted the use of the genes in molecular breeding through BIBAC transformation. Using the BIBACs, we have developed a high-throughput system for functional analysis of the entire Upland cotton genome in the plant model species, *Arabidopsis thaliana*, with which the entire Upland genome could be transformed into *Arabidopsis* within approximately three years by one scientist (M.P. Z, Y.Z, D.M S, and H.-B. Z., unpublished).

Furthermore, the BESs generated in this study provide 9,711 STSs (sequence-tagged sites), with one STS in approximately every 250 kb along the Upland cotton genome. These STSs will facilitate integrative physical and genetic mapping, and sequencing of the Upland cotton genome using the next-generation sequencing technology [47,48]. Using the BIBAC library, we have constructed separate genome-wide physical maps for both the A- and D-subgenomes of Upland cotton cv. TM-1 and identified the minimal tiling path (MTP) clones (15,277 clones) spanning the physical map [46]. We have also initiated a project of sequencing the Upland cotton cv. TM-1 genome based on the BIBAC library using the next-generation sequencing technology (D.M. S., H.-B. Z.). We are developing the integrated physical and genetic maps of individual chromosomes of the Upland cotton genome, with 1–10 contigs per chromosome, from this BIBAC library and a BAC library, which will be necessary for assembling the chromosome-sized pseudomolecules of the Upland cotton genome sequence.

Analysis of the BESs has shown that the 9,711 BESs, with a length range from 50 – 842 bp and an average length of approximately 400 bp, contain about 3,000 exons. If the exons of cotton genes have an average length of 200 – 500 bp [49,50], the 3,000 exons are likely from approximately 3,000 genes, suggesting that approximately 30% of the 9,711 BESs are parts of genes. Among the transposable elements, the LTR retroelement family, *Gypsy/DIRS1*, is the most predominant in the Upland cotton genome, representing over 77% of the transposable elements in the genome. This result is consistent with that of Hawkins et al. [51] estimated for the genomes of its diploid putative progenitor species, *G. raimondii* and *G. herbaceum*, by random shotgun clone sequencing. The more than 1,000 new SSRs (79% of the SSRs) identified from the BESs indicate the abundance of SSR loci in the Upland cotton genome and provide additional tools for cotton genome analysis, and gene and QTL mapping.

The comparative BES analysis has shown that the Upland cotton genome has significantly diverged from the *G. raimondii* genome. The unexpectedly high genome divergence between the two species could be attributed to the rapid evolution of the *G. raimondii* genome, the polyploidization and post-polyploidization evolution of Upland cotton, or both. However, the hypothesis of the rapid evolution of the *G. raimondii* genome does not appear to be compatible with the finding of this study that the A-subgenome of Upland cotton has the same level of similarity as its D-subgenome when compared to the *G. raimondii* genome, even though *G. raimondii* has only a D genome. Therefore, the rapid genome evolution after polyploidization must have occurred in Upland cotton, or both Upland cotton and *G. raimondii*. Moreover, it appears that an extensive element exchange has occurred between the A- and D-subgenomes of Upland cotton during the process of and/or after polyploidization, thus leading to a higher similarity between the two subgenomes at the genomic element sequence level. Therefore, when the A- and D-subgenome specific BESs were aligned to a third genome - the *G. raimondii* genome in this study, similar alignment results were obtained. Zhang et al. [5] studied the gene number variation of nucleotide-binding site (NBS)-encoding gene family and receptor-like kinase (RLK)-encoding gene family between the tetraploid cottons including Upland cotton and their diploid putative progenitor species including *G. raimondii* and *G. herbaceum*. They found that the tetraploid cottons have similar numbers of NBS and RLK genes as their diploid putative progenitor species, suggesting that a large number of genes in the families were lost during the process of and/or after the cotton polyploidizaiton. It has been found recently that most genome-constituent fundamental function elements of rice, including genes, DNA transposable elements, simple sequence repeats and low complexity repeats, have a very low content variation among different chromosomes even though they are non-homologous, suggesting the existence of genomic element exchanges among chromosomes within a genome. These results provide indirect, but strong, support for the above hypothesis. (Liu Y-H, Zhang MP, Wu C, Huang JJ, Zhang H-B: DNA is structured as a linear “Jigsaw Puzzle” in the genomes of *Arabidopsis*, rice and budding yeast, submitted for publication). However, further studies remain to determine the molecular mechanisms underlying the rapid genome evolution and genomic element exchanges between subgenomes in the polyploid cotton cells in the process of post-polyploidization.

## Conclusions

We have constructed a high-quality large-insert plant-transformation-competent BIBAC library for Upland cotton, isolated BIBACs containing several important cotton genes, sequenced nearly 10,000 BESs from BIBACs randomly selected from the cotton BIBAC library and identified over 1,000 new SSR markers from the BESs. These results provide resources and tools useful for advanced research of cotton genomics, genetics and breeding in numerous aspects such as Upland cotton genome integrative physical and genetic mapping, genome sequencing, gene and QTL cloning and characterization, and molecular breeding. Moreover, this study provides some insights into the genome composition and organization of the tetraploid cotton. The retroelement *Gypsy*/*DIRS1* family and the SSRs with (AT) motifs are the most abundant in the Upland cotton genome. Finally, comparative sequence analysis has shown that the genome of the tetraploid Upland cotton has diverged much more than expected from that of its D-subgenome diploid putative progenitor species, *G. raimondii*. The divergence has likely resulted from the rapid evolution of tetraploid cotton and an apparent extensive genome-constituent element exchange or “balancing” between the A- and D-subgenomes of the tetraploid cotton during the process of post-polyploidization, even though further studies remain to test this hypothesis.

## Methods

### Plant materials

Upland cotton cv. TM-1 was used as the source DNA of the BIBAC library. The seeds of the cultivar were obtained from USDA/ARS, College Station, Texas, germinated and grown in a growth chamber at 25°C, 16 h light/8 h dark. Plant seedlings were treated in continuous dark for 48 h immediately before DNA isolation to minimize polyphenolics and polysaccharides. Cotyledons were harvested, frozen in liquid nitrogen and stored at -80°C before use.

### Preparation of megabase-sized nuclear DNA

Megabase-sized DNA was prepared according to Zhang et al. [37] with minor modifications [39,40,42,52]. Nuclei were washed for two additional times to minimize the contamination of polyphenolics, polysaccharides, chloroplasts and mitochondria contained in cytoplasm. The nuclei suspension of approximately 2 × 10^7^ nuclei /ml was prepared and used to make low-melting-point (LMP) agarose plugs. Therefore, each 100 μl-plug contained approximately 5 μg nuclear DNA.

### Preparation of BIBAC vector

The BIBAC vector pCLD04541 [27] was used for the construction of the BIBAC library. This vector has been widely used in the construction of BIBAC libraries for a number of species [22,28-35]. Vector DNA was isolated by the alkaline lysis method and purified by two rounds of the cesium chloride gradient centrifugation [38,53]. The vector DNA was completely digested with *Bam*H I and prepared according to a procedure that we developed previously [38,42].

### Library construction

The nuclear DNA of Upland cotton was partially digested with *Bam*H I and cloned in the BIBAC vector pCLD04541 to construct the BIBAC library [38]. To determine the optimal condition for partial digestion of megabase-sized DNA in LMP agarose plugs, particularly the amount of restriction enzyme (*Bam*H I) per reaction and digestion incubation time, we first conducted a partial digestion test using a series of *Bam*H I concentrations per reaction. From the results of the partial digestion test, 2.4 units of *Bam*H I per reaction containing one-third of a 100-μl megabase-sized DNA plug and 8-min incubation at 37°C were selected for large-scale partial digestion for the library construction.

Ten 100-μl megabase-sized DNA plugs were used for partial digestion of the library construction using the condition predetermined by the partial digestion test (2.4 units of *Bam*H I per reaction and 8-min incubation at 37°C). The partially digested megabase-sized DNA was subjected to one size selection on a 1% agarose gel by pulsed-field gel electrophoresis and DNA fragments ranging from 100–250 kb were selected [38]. The selected DNA fragments contained in the agarose gel were electroeluted, dialyzed against 0.5 × TE (5 mM Tris–HCl, 0.5 mM EDTA, pH 8.0) and ligated into the dephosphorylated pCLD04541 vector. The ligation was performed at a molar ratio of 3 vectors: 1 insert, 1.5 ng of insert DNA /μl of ligation reaction and 1.0 unit T4 DNA ligase (Invitrogen) /50 μl of ligation reaction at 16°C for 8 h.

The ligated DNA was transformed into *Escherichia coli* strain DH10B (Invitrogen) by electroporation using the Cell Porator™ Device (Gibco BRL, Carlsbad, CA) consisting of Power Supply (cat. 1600), Chamber Safe (cat. 1600) and Voltage Booster (cat. 1612). The electroporation settings were as Zhang et al. [38], and 375 volts were used for electroporation. The transformed cells were selected for transformants and recombinant BIBACs on selective LB agar plates containing 15 μg of tetracycline, 15 μg of IPTG (isopropylthiogalactoside) and 60 μg of X-Gal (5-bromo-4-chloro-3-indolyl-β-D-galactoside) per ml of medium. Ligation selection and large-scale transformation for library construction were described as Zhang et al. [38]. The recombinant white clones were arrayed as individual BIBACs in 384-well microtiter plates, duplicated and kept at -80°C.

### Library characterization

A random sample of the clones arrayed in the 384-well microtiter plates was analyzed [38] to estimate the insert sizes of the library clones and the percentage of clones having no inserts (insert-empty clones). BIBAC DNA was isolated, digested with *Not* I to release the insert of cotton DNA from the cloning vector pCLD04541 and subjected to pulsed-field gel electrophoresis. The insert size of each clone was estimated by the sum of the sizes of all insert band(s) in each gel lane using the lambda ladder PGE marker (New England BioLabs, Ipswich, MA) as the molecular weight marker.

The BIBAC library was printed in a 4 × 4 format using the GeneTAC Robotic Workstation (Genomic Solutions Inc., Ann Arbor, MI) onto 22.5 × 22.5 cm Hybond N + membrane (Amersham-Pharmacia, Piscataway, NJ) laid on LB agar medium containing 15 μg tetracycline/ml medium. To facilitate reading the positive clones accurately and minimize the false positive clones, each of the library clones was printed in duplicate on the membrane. Therefore, each 22.5 × 22.5 membrane contained a total of 18,432 (48 × 384) double-printed clones and the entire library was printed on four 22.5 × 22.5 cm and one 12 × 8 cm membranes. The clones on the membranes were grown at 37°C overnight, the membranes were processed and the DNA of each clone was cross-linked on the membranes [41,54]. The membranes were hybridized with a probe prepared from a mixture of three chloroplast genes [41], *ndh*A, *rbc*L and *psb*A, to identify the clones of the library that were derived from chloroplast DNA. To ensure unambiguously reading the positive clones identified on the library membranes, we added 1–2 ng [1 - 2% (w/w) of the probe DNA] of the pCLD04541 vector DNA to the labeling reaction to create appropriate background when hybridized. The hybridization was done at 65°C overnight. After hybridization, the library membranes were washed in 1 × SSC, 0.1% (w/v) SDS [54] at 65°C twice, 10 min each time, followed by 0.5 × SSC, 0.1% SDS at 65°C twice, 10 min each time.

### Identification of BIBACs containing genes important to cotton

The membranes of the BIBAC library were screened to further verify its quality, demonstrate its utility and to identify the BIBACs containing cotton genes of interest. We first hybridized the library membranes using gene-specific overgo probes to identify the BIBACs containing the genes involved in fiber development (*MYBB*, *MYBT2* and *RDL1*), fiber cellulose biosynthesis (*CelA1*, *CelA3*, *CelA6*, *GhCesA2*, *GhIRX3*, *GhCesA3* and an unnamed *Ces*), seed fatty acid metabolism (*FADO6*) and cotton-nematode interaction (*MIC3* and *MIC1-15*; Additional file 1: Table S1). The gene-specific overgos were designed from the unique sequences of the target genes, synthesized, pooled at equal amounts (100 ng per overgo pair) and used as probes to hybridize the library membranes [34]. The positive clones resulted from the primary hybridization were then re-arrayed into a new microtiter plate, double-printed onto a 12 × 8 cm Hybond N + membrane in a 2 × 2 format and sorted according to individual gene overgos using a 4 × 4 pooling hybridization strategy [55,56]. To ensure unambiguously reading the positive clones identified on the library membranes, we added 1–2 ng [1 - 2% (w/w) of the gene overgos] of pCLD04541-specific overgos (forward oligo 5’-TTAAGTTGGGTAACGCCAGGGTTT-3 and reverse oligo 5’-CAACGTCGTGACTGGGAAAACCCT-3’) to the gene-specific overgos. The gene and vector overgo mixture was labeled with [^32^P]-dATP and [^32^P]-dCTP to increase the hybridization signals, and the hybridization was carried out at 60°C for overnight. After hybridization, the library membranes were washed in 1 × SSC, 0.1% (w/v) SDS at 60°C twice, 10 min each time, followed by 0.5 × SSC, 0.1% SDS at 60°C twice, 10 min each time.

Moreover, we also hybridized the library membranes to identify the BIBACs containing the disease resistance analogues (RGAs) from three loci, 1C08 (GenBank No.: AY600376), 2D14 (AY600383), and 2D03 (AY600401)/2B19 (AY600394) [43]. The 1C08 RGA shares a significant similarity with the tomato root-knot nematode resistance gene, *Mi-1.2*[43,57] and is closely linked to the *B2* gene of the cotton bacterial blight (*X. campestris* pv. *malvacearum*) resistance [43,44]. The 2D14 and 2D03/2B19 RGAs flank the Qb_6a_ QTL of the bacterial blight resistance [43,44]. The inserts of the RGA clones were excised by enzymatic digestion and gel purification, labeled with [^32^P]-dCTP using the random priming method and used as probes to hybridize the library membranes as the above library hybridization with the chloroplast gene probes.

### BIBAC end (BES) sequencing and analysis

The BIBAC ends were sequenced according to Li et al. [58] with a few modifications using the Sanger method. Random BIBACs were inoculated into 96-deep well blocks containing 1.0 ml of Terrific Broth medium per well and grown overnight in an incubator at 37°C, 300 rpm. DNA was extracted using the alkaline lysis method [53] with modifications [59]. The DNA was dissolved in 15 μl 0.5 × TE. The sequencing reaction included 2 μl BigDye Terminator v3.1 Cycle, 2 μl 5 × Sequence Buffer (Applied Biosystems, Carlsbad, CA), 300–500 ng of template BIBAC DNA, 0.4 μl 50 μmol/L primer (PCLD04541_T7: 5^′^-TAATACGACTCACTATAGGG-3^′^ or PCLD04541_Rev primer: 5^′^-GAAAAGCTGGTACGTA-3^′^) and distilled water added to 10 μl. The sequencing reaction was conducted by PCR at 95°C for 4 min, followed by 99 cycles of 95°C for 15 s, 46°C for 10 s and 60°C for 4 min. The sequencing reaction was purified by isopropanol precipitation, followed by two washes with 70% (v/v) ethanol. Sequencing was carried out on ABI 3100 Genetic Analyzer (Applied Biosystems, Carlsbad, CA).

Software Phred [60,61] was used for sequence base calling, with a quality score of Q20. The vector sequences were removed using Cross_match (http://www.phrap.org/phredphrapconsed.html#block_phrap). Sequence trimming was conducted by Sequencher v. 3.7 (Gene Codes Corp., Ann Arbor, MI). The repeat elements in the BESs were identified using RepeatMasker (A. Smit, G. Glusman and R. Hubley at http://repeatmasker.org) against the latest repeat library (as of 04/19/2011) of *Arabidopsis thaliana* that is the phylogenetically closest to cotton in the database, with the default parameters. We also predicted the genes/exons contained in the BESs using Genscan [62] and GeneMark [63] against *Arabidopsis* model/smat using the default settings. The BESs were searched against the recently released *G. raimondii* genome sequence assembly (DOE Joint Genome Institute: Cotton D V1.0, http://www.phytozome.net/cotton.php) by a local Blat server [64] with a continuously minimal match of 100 bp and a sequence identity of 100%, 95%, 90%, 80%, 70% and 60%, respectively. Microsatellites were identified in non-redundant BESs using Msatfinder v2.0 that was specifically designed to identify and characterize microsatellites [65]. Only the microsatellites of 2–6 nucleotide motifs with at least 4, 5 and 6 repeat units were collected.

## Abbreviations

BIBAC: Binary bacterial artificial chromosome; BAC: Bacterial artificial chromosome; BES: BIBAC or BAC end sequence; SSR: Simple sequence repeat; RGA: Resistance gene analogue; EST: Expressed sequence tag; STS: Sequence-tagged site; QTL: Quantitative trait locus

## Competing interests

The authors declare that they have no competing interests.

## Authors’ contributions

H-BZ conceived, designed and guided the study, and edited the manuscript. M-KL, YZ and MPZ performed experiments, including library construction, characterization, BES sequencing and analysis, and comparative sequence analysis. YZ wrote the manuscript while M-KL and MPZ edited the manuscript. HJK and BAT screened the library with some *Cel* genes and edited the manuscript. MG helped assemble and characterize the library. DMS guided the study and assisted in characterization of the library. All authors read and approved the final manuscript.

## Supplementary Material

Additional file 1: Table S1 Gene-specific overgos used for the Upland cotton cv. TM-1 BIBAC library screening. Click here for file
