# Supplementary material for: Construction of a plant-transformation-competent BIBAC library and genome sequence analysis of polyploid Upland cotton (Gossypium hirsutum L.)
Source: BMC Genomics. 2013 Mar 28;14:208. doi: 10.1186/1471-2164-14-208 (PMC3623804; doi:10.1186/1471-2164-14-208)
Supplement: Additional file 1: Table S1 — Gene-specific overgos used for the Upland cotton cv. TM-1 BIBAC library screening. [file 1471-2164-14-208-S1.pptx]

## Slide 1
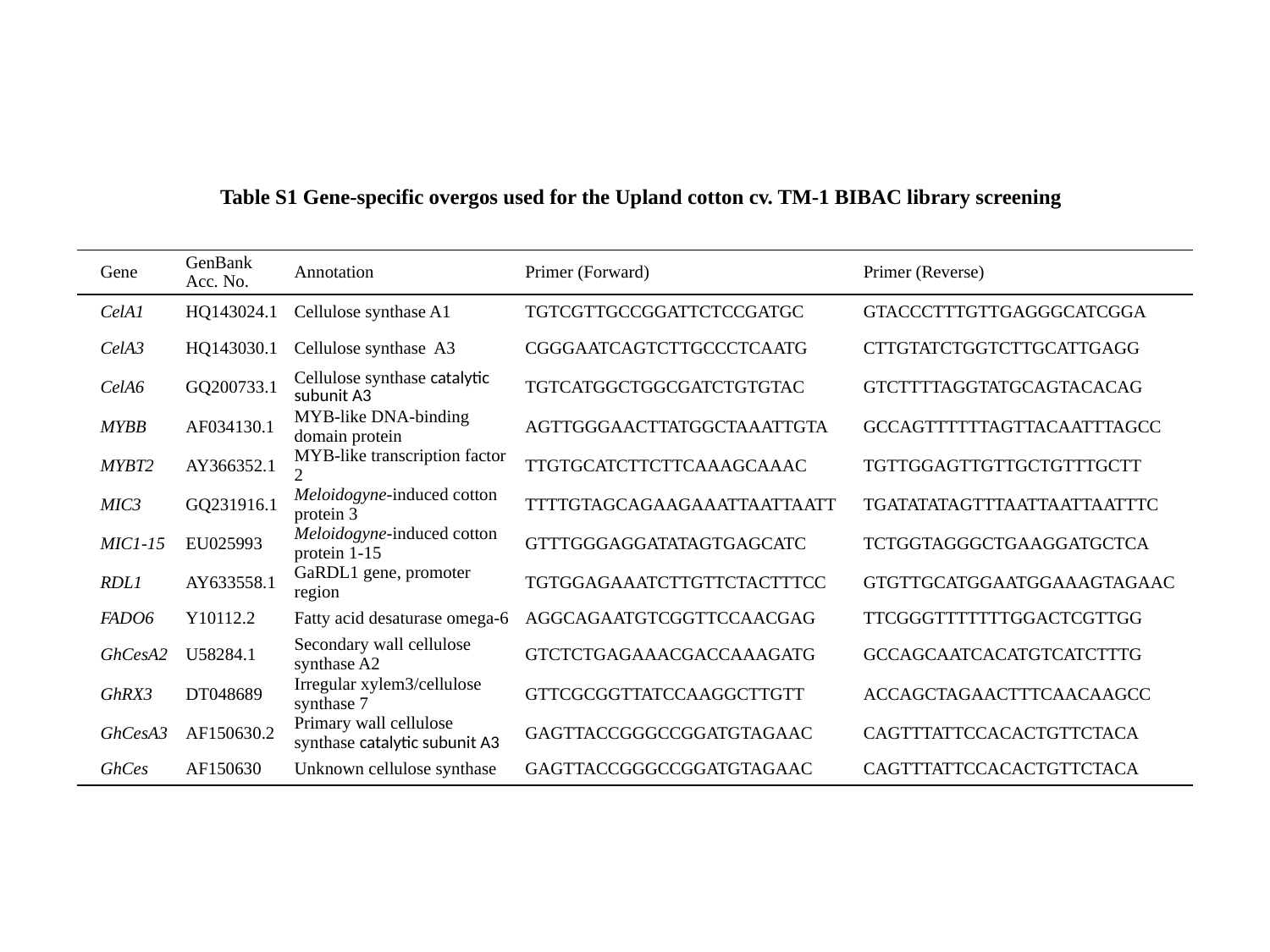

Table S1 Gene-specific overgos used for the Upland cotton cv. TM-1 BIBAC library screening
| Gene | GenBank Acc. No. | Annotation | Primer (Forward) | Primer (Reverse) |
| --- | --- | --- | --- | --- |
| CelA1 | HQ143024.1 | Cellulose synthase A1 | TGTCGTTGCCGGATTCTCCGATGC | GTACCCTTTGTTGAGGGCATCGGA |
| CelA3 | HQ143030.1 | Cellulose synthase A3 | CGGGAATCAGTCTTGCCCTCAATG | CTTGTATCTGGTCTTGCATTGAGG |
| CelA6 | GQ200733.1 | Cellulose synthase catalytic subunit A3 | TGTCATGGCTGGCGATCTGTGTAC | GTCTTTTAGGTATGCAGTACACAG |
| MYBB | AF034130.1 | MYB-like DNA-binding domain protein | AGTTGGGAACTTATGGCTAAATTGTA | GCCAGTTTTTTAGTTACAATTTAGCC |
| MYBT2 | AY366352.1 | MYB-like transcription factor 2 | TTGTGCATCTTCTTCAAAGCAAAC | TGTTGGAGTTGTTGCTGTTTGCTT |
| MIC3 | GQ231916.1 | Meloidogyne-induced cotton protein 3 | TTTTGTAGCAGAAGAAATTAATTAATT | TGATATATAGTTTAATTAATTAATTTC |
| MIC1-15 | EU025993 | Meloidogyne-induced cotton protein 1-15 | GTTTGGGAGGATATAGTGAGCATC | TCTGGTAGGGCTGAAGGATGCTCA |
| RDL1 | AY633558.1 | GaRDL1 gene, promoter region | TGTGGAGAAATCTTGTTCTACTTTCC | GTGTTGCATGGAATGGAAAGTAGAAC |
| FADO6 | Y10112.2 | Fatty acid desaturase omega-6 | AGGCAGAATGTCGGTTCCAACGAG | TTCGGGTTTTTTTGGACTCGTTGG |
| GhCesA2 | U58284.1 | Secondary wall cellulose synthase A2 | GTCTCTGAGAAACGACCAAAGATG | GCCAGCAATCACATGTCATCTTTG |
| GhRX3 | DT048689 | Irregular xylem3/cellulose synthase 7 | GTTCGCGGTTATCCAAGGCTTGTT | ACCAGCTAGAACTTTCAACAAGCC |
| GhCesA3 | AF150630.2 | Primary wall cellulose synthase catalytic subunit A3 | GAGTTACCGGGCCGGATGTAGAAC | CAGTTTATTCCACACTGTTCTACA |
| GhCes | AF150630 | Unknown cellulose synthase | GAGTTACCGGGCCGGATGTAGAAC | CAGTTTATTCCACACTGTTCTACA |
